# One-step fabrication of lidocaine/CalliSpheres^®^ composites for painless transcatheter arterial embolization

**DOI:** 10.1186/s12967-022-03653-8

**Published:** 2022-10-11

**Authors:** Chuan Tian, Zijian Wang, Lei Huang, Yimin Liu, Kunpeng Wu, Zhaonan Li, Bin Han, Dechao Jiao, Xinwei Han, Yanan Zhao

**Affiliations:** 1 Department of Interventional Radiology, The First Affiliated Hospital of Zhengzhou University, Zhengzhou, 450052 China; 2 Department of Urology, Zhongnan Hospital of Wuhan University, Wuhan, 430071 China; 3 Department of Plastic Surgery, Zhongnan Hospital of Wuhan University, Wuhan, 430071 China; 4 Department of Radiotherapy, The First Affiliated Hospital of Zhengzhou University, Zhengzhou, 450052 China

**Keywords:** CalliSpheres^®^ bead, Lidocaine, Drug delivery, Anti-tumor, Inflammation micro-environment

## Abstract

**Background:**

Transcatheter arterial embolization (TAE) is one of the first-line treatments for advanced hepatocellular cancer. The pain caused by TAE is a stark complication, which remains to be prevented by biomedical engineering methods.

**Methods:**

Herein, a commercial embolic agent CalliSpheres^®^ bead (CB) was functionally modified with lidocaine (Lid) using an electrostatic self-assembly technique. The products were coded as CB/Lid-n (n = 0, 5, 10, corresponding to the relative content of Lid). The chemical compositions, morphology, drug-loading, and drug-releasing ability of CB/Lid-n were comprehensively investigated. The biocompatibility was determined by hemolysis assay, live/dead cell staining assay, CCK8 assay, immunofluorescence (IHC) staining assay and quantitative real-time PCR. The thermal withdrawal latency (TWL) and edema ratio (ER) were performed to evaluate the analgesia of CB/Lid-n using a plantar inflammation model. A series of histological staining, including immunohistochemistry (IL-6, IL-10, TGF-β and Navi1.7) and TUNEL were conducted to reveal the underlying mechanism of anti-tumor effect of CB/Lid-n on a VX2-tumor bearing model.

**Results:**

Lid was successfully loaded onto the surface of CalliSpheres^®^ bead, and the average diameter of CalliSpheres^®^ bead increased along with the dosage of Lid. CB/Lid-n exhibited desirable drug-loading ratio, drug-embedding ratio, and sustained drug-release capability. CB/Lid-n had mild toxicity towards L929 cells, while triggered no obvious hemolysis. Furthermore, CB/Lid-n could improve the carrageenan-induced inflammation response micro-environment in vivo and in vitro. We found that CB/Lid-10 could selectively kill tumor by blocking blood supply, inhibiting cell proliferation, and promoting cell apoptosis. CB/Lid-10 could also release Lid to relieve post-operative pain, mainly by remodeling the harsh inflammation micro-environment (IME).

**Conclusions:**

In summary, CB/Lid-10 has relatively good biocompatibility and bioactivity, and it can serve as a promising candidate for painless transcatheter arterial embolization.

**Supplementary Information:**

The online version contains supplementary material available at 10.1186/s12967-022-03653-8.

## Introduction

Hepatocellular carcinoma (HCC) is one of the most common cancers worldwide, with an annual incidence rate and mortality rate of 8.3 × 10^5^ and 4.1 × 10^5^, respectively [1, 2]. Affected by hepatitis virus, metabolic dysfunction and multi nidus, no more than 30% of HCC patients can receive standard surgical resection [3]. In recent decades, minimally invasive interventional therapy (MIIT) has emerged as a promising substitute [4, 5]. Transcatheter arterial embolization (TAE) is highly recommended for advanced HCC patients who have no indications for surgical resection or liver transplantation [6, 7]. The Japan Society of Hepatology (JSH) also nominated TAE as the golden standard of advanced HCC [8]. The median survival time of HCC patients treated by TAE was 3.1 years, and the 2-year overall survival rate was 75%, which was relatively satisfactory [9]. A series of embolic agents, including lipiodol, gelatin sponges, polyvinyl alcohol (PVA) microspheres and drug-loaded composites, have been successfully developed [10, 11]. These embolic agents can swell to block blood flow, and sustained release bioactive drugs to specific tumor cells. Currently, the commonly used embolic agents are centralized supplied by several transnational corporations. With the spread of diagnosis related groups (DRGs) in China, domestic products with better performance and lower cost will be more competitive.

CalliSpheres^®^ bead (CB) is the first domestic embolic microsphere [12]. CB is prepared with medical grade PVA, and then its surface is modified by negatively charged component to enhance the drug-loading capability. A series of CB with different size and drugs have been on the market to meet complex clinical needs [12, 13]. Notably, the clinical effectiveness and safety of CB were both approved by the U.S. Food and Drug Administration (FDA) and the National Medical Products Administration (NMPA) of China. Our group is one of the earliest medical institutions, which use CB to treat advanced HCC patients. We found that post-operative pain was a stark complication of TAE. In this case, pain sensation could be attributed to the acute ischemia and necrosis of solid tumor, cell debris-induced inflammatory, and abnormal tissue tension [14–16]. To relieve pain, these patients are conventionally given oral nonsteroidal anti-inflammatory drugs (NSAID) or opioids [17]. Unfortunately, the side effects of NSAID and opioids, such as digestive hemorrhage, liver injury, and drug addiction, frequently lead to the failure of antalgic therapy [18]. Up to now, tumor-targeted drug delivery is of increasing interest from bench to bedside. However, the drug-loaded CB with analgesic effect has rarely been developed.

Lidocaine (Lid) is an amide anesthetic, which has better local analgesic effect and biosafety than NSAID and opioids [19]. Lid has been applied to painless TAE treatment for a long time. Massimo reported that the pain scores of HCC patients were significantly decreased by injecting Lid into intrahepatic artery [20, 21]. These results owned to that Lid improved the peripheral ectopic discharge threshold, and reduced the ectopic discharge of nociceptive nerve fibers by blocking sodium channels (Nav1.7, Nav1.8) [22]. Meanwhile, Lid could remodel the harsh inflammation micro-environment (IME) to inhibit the release of endogenous pain-inducing factors, such as neurotransmitters, bradykinin, tetracosane, neurotrophins, cytokines, chemokines, and lipids (prostaglandins, thromboxane, leukotrienes, endocannabinoids) [22–24]. According to previous literatures, Lid has been applied for IME-targeted therapy of airway disease, and its’ molecular mechanism was largely uncovered [25]. Thus, we inferred that Lid could also inhibit the TAE-induced pain among advanced HCC patients, through analgesia and IME-remodeling pathway at the same time.

**Fig. 1:**
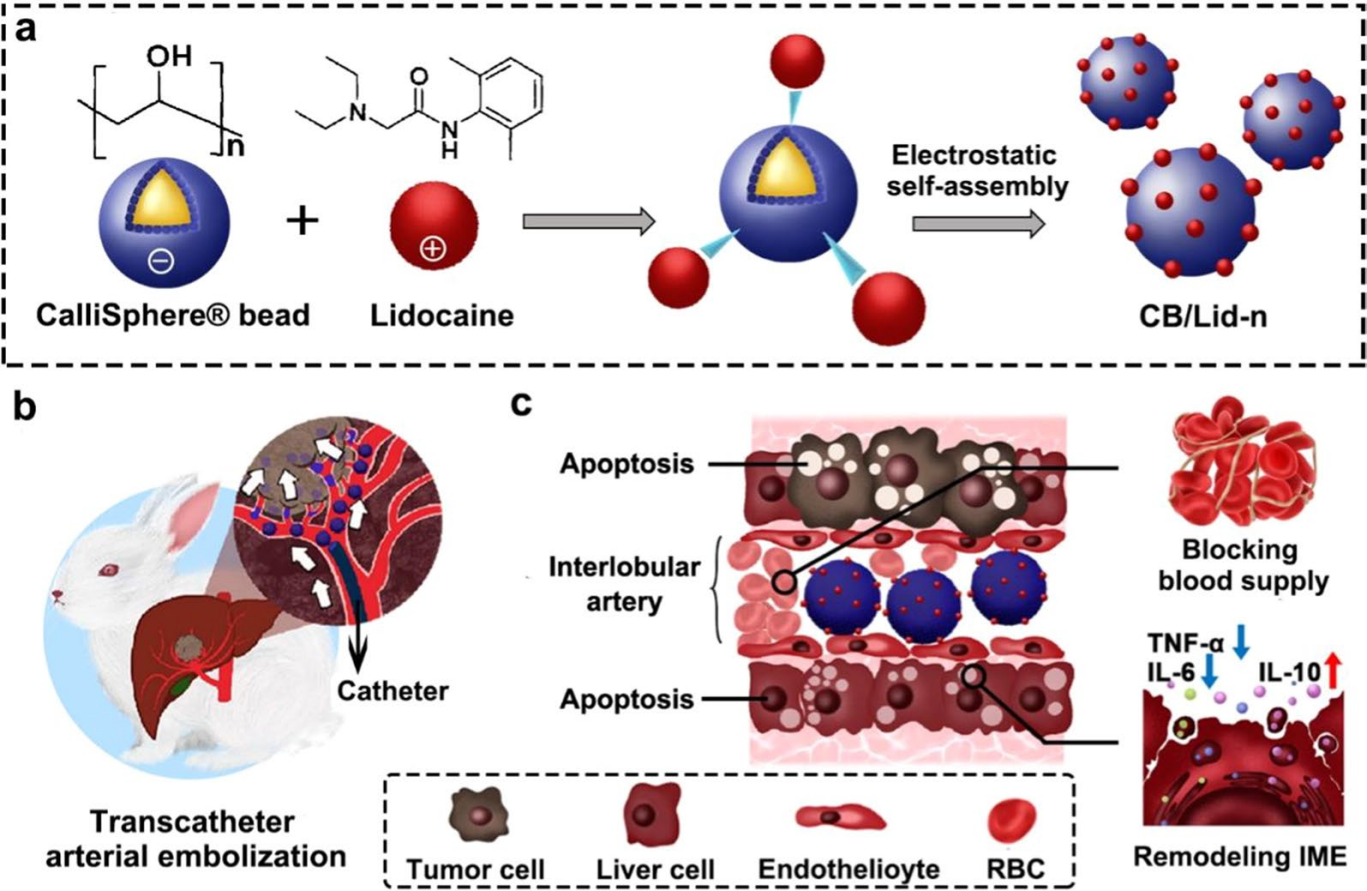
Schematic illustration of the lidocaine/CalliSpheres^®^ composites. **a** the negatively charged CalliSpheres^®^ bead was electrostatically self-assembled with the positively charged lidocaine to fabricate drug-loaded composites (CB/Lid-n); **b** CB/Lid-n was used for painless transcatheter aterial embolization in a rabbit VX2-tumor bearing model; **c** CB/Lid-n could block blood supply and remodel harsh inflammation micro-environment (IME)

Herein, a series of Lid-loaded CB composites (named as CB/Lid-n) were fabricated. A schematic diagram of this work is shown in Fig. 1. Lid was positively charged, and then loaded onto the negatively charged CB using an electrostatic self-assembly (ESA) technique. ESA is more feasible and biocompatible than chemical cross-linking [26, 27]. CB/Lid-n was characterized by a series of tests in vitro, such as scanning electron microscope (SEM), CCK8 assay and hemolysis assay. The application potential of CB/Lid-n in vivo were further evaluated using the plantar inflammation model and VX2-tumor bearing model. It was hypothesized that CB/Lid-n had relatively good drug-loading and drug-releasing capability, cytocompatibility and hemocompatibility, anti-inflammation and anti-tumor effects. This work is of great significance for fabricating transformable medical devices to promote clinical progress.

## Materials and methods

### Materials

CalliSpheres® were kindly obtained from Hengrui Medical Co., Ltd. (Jiangsu, China). Lidocaine hydrochloride was purchased from Meryer Chemical Co., Ltd. (Shanghai, China). Polyvinyl alcohol (PVA) was provided by Macklin Biotech Co., Ltd. (Shanghai, China). Recombinant mouse IL-6 protein was purchased from Abbkine. Co., Ltd. (Wuhan, China). Anti IL-10 receptor-blocking monoclonal antibody was purchased from Bx-cell Co., Ltd. (Lebanon, USA). Mouse lung fibroblasts (L929) was obtained from the Medical Research Center, Zhongnan Hospital of Wuhan University. RPMI-1640 culture medium, fetal bovine serum, antibiotic solution, phosphate buffer saline (PBS), and trypsin solution were purchased from Thermo Fisher Scientific Co., Ltd. (Shanghai, China). Dimethyl sulfoxide (DMSO), paraformaldehyde (PFA), solid paraffin, sodium chloride, and isoflurane were purchased from Sinopharm Chemical Reagent Co., Ltd. (Shanghai, China). Other chemical and biological reagents were purchased and used without purification.

### Fabrication of lidocaine/CalliSpheres^®^composites

A one-step ESA technique were used to fabricate the lidocaine/CalliSphere^®^ composites. Briefly, lidocaine hydrochloride and commercial CalliSphere^®^ beads were dissolved into phosphate buffer saline with a pH value of 6.0. After that, they were mixed and electrostatically self-assembled using a three-way pipe. Table 1 shows the codes (CB/Lid-n, n = 0, 5 and 10) and compositions of lidocaine/CalliSpheres^®^ composites, in which CB represents CalliSpheres^®^ beads, Lid represents lidocaine, and n represents the volume of lidocaine solution. The obtained CB/lid-n samples were centrifuged to remove the supernatant, and then resuspended into normal saline for further study.

**Table 1:** The codes and compositions of lidocaine/CalliSpheres^®^ composites

Sample codes	CalliSpheres^®^ beads (g)	2% Lidocaine solution (mL)
CB/Lid-0	2.0	0.0
CB/Lid-5	2.0	5.0
CB/Lid-10	2.0	10.0

### Physicochemical characterizations

The morphology of CB/Lid-n was captured using a scanning electron microscopy (JSM-7401 F, JEOL, Japan) installed with an energy dispersive spectrum (EDS) analysis system [28]. Before tests, the samples were coated with gold for 60 s in a vacuum environment. The diameter of CB/Lid-n was measured using an Image J software [29]. At least 50 independent samples were used for statistical analysis.

### Drug-loading efficiency of CB/Lid-n

Lid (2.5 mg) was added into 25 mL volumetric flask to obtain 100 μg/mL Lid solution, and then step-by-step diluted to prepare solution with concentrations of 2, 4, 8, 16, 20 and 40 μg/mL, respectively. The standard curve of Lid was measured by detecting their absorbance value at 262 nm using a microplate Reader (SuPerMax 3100, Shanpu, China). CB (0.2 g) were added into 0, 0.5 and 1.0 mL of Lid solution, respectively. After incubation for 2, 5, 10, 30 and 60 min, the samples were centrifuged at 12,000 rpm for 1 min, and the absorbance value of supernatants was determined at 262 nm. The concentration of Lid is converted by its standard curve. The drug-loading ratio and drug-embedding ratio were measured.

### Drug-releasing dynamics of CB/Lid-n

Drug-loaded CB/Lid-n samples (0.2 g) were sealed into a dialysis bag (MW = 3500) and then immersed into 20 mL PBS. All the samples were constantly stirred at 37 ℃ with a speed of 100 rpm. At regular time intervals, the absorbance value of supernatants was determined at 262 nm, and then converted into the concentration of Lid using the standard curve. The drug releasing dynamics of CB/Lid-n were measured from three independent samples.

### Hemolysis assay

This study was approved by the animal care committee, the First Affiliated Hospital of Zhengzhou University (Approval Number: 2021-19), and carried out according to the National Institute of Health’s guideline for the care and use of laboratory animals. Healthy New Zealand rabbits were obtained from the Hualan Biological Co., Ltd. (Henan, China), and then anesthetized by isoflurane inhalation. Fresh whole blood was collected into an anticoagulant tube containing 3.8% sodium citrate solution, and then diluted with normal saline at a ratio of 1:1.25. 0.2 g CB/Lid-n samples and 10 mL normal saline were added into 15 mL tubes, followed by adding 0.2 mL diluted blood. According to our previous work, a positive control group and a negative control group were also set [30]. All the samples were incubated at 37 ℃ for 60 min, and then centrifuged at 1000 rpm for 10 min. Finally, the absorbance value of supernatants was determined at 545 nm using a microplate reader. The hemolysis rate (HR) of CB/Lid-n was calculated as follows:

$$\mathrm{HR }\left(\mathrm{\%}\right)=\frac{\mathrm{Ac}-\mathrm{An}}{\mathrm{Ap}-\mathrm{An}}*100$$

In which, Ap, An and Ac represented the absorbance value of positive control group, negative control group, and CB/Lid-n group, respectively [31].

### Cytocompatibility evaluations

#### Live/dead cell staining assay

The extracts of CB/Lid-n were prepared according to the ISO10993-12:2007. The viability of L929 cells was characterized by a live/dead cell staining assay [32]. The L929 cells were seeded onto 24-well plates at a density of 5 × 10^4^ per well for 24 h. The cultured medium was replaced with 125 μL extracts and 225 μL fresh CCM, and then incubated for another 72 h. The samples were rinsed with PBS for 3 time, and then incubated with 1 mL dye containing 1 µL AM and 0.5 µL PI at room temperature for 30 min. All the samples were rinsed again, and then captured with an inverted fluorescence microscope (IXplore Standard, Olympus, Japan). At least five random fields are captured.

#### CCK8 assay

This study was performed with a modified transwell chamber assay. L929 cells were seeded onto the lower chamber with suitable cell density, and CB/Lid-n was added onto the upper chamber. At regular time intervals (1, 2 and 3 days), appropriate CCK8 solution was added into each well, then incubated for another 2 h. The absorbance value of each well was detected at 450 nm. At least six independent samples were used for statistical analysis.

#### Immunofluorescence staining assay

L929 cells were incubated with 2% carrageenan-containing culture medium for 24 h, and then treated with the extracts of CB/Lid-n for another 24 h. After fixed with 4% PFA solution for 15 min, immunofluorescence (IF) staining was performed according to the standard protocols. Briefly, the samples were blocked by goat serum for 30 min, labeled by primary antibody overnight, and re-labeled by fluorescent secondary antibody for 1 h. The primary antibody and secondary antibody used in this work are listed in Additional file 1: Table S1. The nucleus was visualized by DAPI. The IF images of the treated cells were captured using a laser confocal microscope (Multiplex SR-2Y, ZEISS, Germany).

### Gene detection

The treated L929 cells obtained in “Immunofluorescence staining assay” were lysed for quantitative real-time PCR (qRT-PCR) analysis. cDNA samples were prepared using a 2-step reverse transcription process, and then applied for the qRT-PCR in accordance with the standard protocols [33]. The primer sequences used in thizus work are listed in Additional file 1: Table S2.

### *Analgesia evaluations *in vivo

The pain-relief effect of CB/Lid-n was evaluated using a plantar inflammation model. 24 BALB/c mice were purchased from the Hualan Biological Co., Ltd. (Henan, China), and randomly divided into 4 groups. 100 µL carrageenan solution was injected subcutaneously into the plantar of the left hind toe of each animal. Meanwhile, 100 µL CB/Lid-n (n = 0, 5, 10) solution with a concentration of 0.2 mg/mL was injected into the same sites, and the mixed solution of CB/Lid-n was nearly neutral. The blank control group was treated with equivalent normal saline. After operations, all the animals were fed in a specific pathogen free (SPF) environment for further study.

At regular time intervals, thermal withdrawal latency (TWL) of the injured limbs was detected by infrared irradiation. The experimental protocols referred to previous literature with some modifications [34]. The base value of TWL was adjusted to 10–12 s. At least 3 independent tests were performed in each group. Plantar thickness was also measured using a vernier caliper. Edema ratio (ER) of plantar thickness was calculated as follows:

$$\mathrm{GR }\left(\mathrm{\%}\right)=\frac{\mathrm{Tn}}{\mathrm{T}0}*100$$

In which, T0 and Tn represented the plantar thickness at day 0 and day n, respectively.

At day 1 and day 3, fresh toe tissues were resected, and fixed with 4% PFA solution for 48 h. HE, Masson, toluidine blue, and immunohistochemical staining assay (Navi1.7, IL-6, and IL-10) were performed according to the standard protocols. The primary antibody and secondary antibody used in this work are listed in Additional file 1: Table S1. The histological images were captured using an inverted fluorescence microscope. Image J software was used for quantitative analysis.

To identify the mechanism of anti-inflammation activity of CB/Lid-10, a rescue assay was supplied. The plantar inflammation model was constructed as described before. Blank control group was treated with 100 normal saline, CB/Lid-0 and CB/Lid-10 groups were respectively treated with equivalent 0.2 mg/mL CB/Lid-0 and CB/Lid-10 solution. To block the anti-inflammatory activity of CB/Lid-10, recombinant IL-6 protein and anti-IL-10 receptor-blocking monoclonal antibody was injected in accordance with previous report [35–37], and named as CB/Lid-10 + IL6 group and CB/Lid-10 + anti IL10 group. At regular time intervals, the TWL and ER of the injured limbs were detected.

### Anti-tumor evaluations using a VX2-tumor bearing model

#### Construction of animal model

Thirty male New Zealand rabbits were purchased from the Hualan Biological Co., Ltd. (Henan, China), and randomly divided into 3 groups. All the animals were anesthetized by isoflurane inhalation, and their abdomen were operated to expose the liver. Fresh VX2 tumor tissues were cut into 1 mm^3^ squares, and then transplanted into the livers using a puncture needle. All the animals were sutured, and treated by antibiotic injection for 3 days. The animals were fed in SPF environment for 2 weeks. After that, doppler ultrasonography and enhanced computed tomography were performed to identify the construction of tumor bearing animals.

#### Transcatheter arterial embolization

Transcatheter arterial embolization (TAE) were carried out. Briefly, a 2 cm incision was made at the right groin to expose the femoral artery. The 5-F catheter was inserted into the femoral artery, followed by the 2.7-F microcatheter and 0.021 inches guide wire. The microcatheter moved to the tumor blood supply artery, and released 500 µL normal saline containing 100 mg CB/Lid-0 or CB/Lid-10. The blank control group were treated with equivalent normal saline without CB/Lid-n. After that, the microcatheter, guide wire and catheter were removed, and the femoral artery was sutured. All the animals were given antibiotic injection for 3 days to avoid bacterial infection.

### Data acquisition

2 mL anticoagulant hepatic arterial blood was collected at 1, 3, 5, 15 and 30 min after embolization, and then centrifugated at 3000 rpm for 15 min. The concentration of Lid in serum was detected by a liquid chromatograph mass spectrometer (HP-LC. LC-20AD, Agilent, China). At regular time intervals, the animals were executed, and the liver and tumor tissues were cut and photographed. A series of histological staining, including HE, immunohistochemistry (IL-6, IL-10, TGF-β and Navi1.7), and TUNEL (Ki67) were also performed. The histological images were captured using an inverted fluorescence microscope. Image J software was used for quantitative analysis.

### Statistical analysis

SPSS 20.0 software (IBM, USA) incorporated with T test and one-way ANOVA was used for statistical analysis. *P* < 0.05 indicated significant difference.

## Results and discussion

### Preparation and characterization of CB/Lid-n

In this study, a series of lidocaine/CalliSpheres^®^ composites were firstly prepared for painless transcatheter arterial embolization (TAE). Commercial CalliSpheres^®^ beads (CB) are negatively charged [10], and can electrostatically self-assembled with the positively charged lidocaine (Lid) under slightly acidic environment. Notably, we can easily manipulate the drug-loading, sustained-releasing and other biological activities by adjusting the proportion of CB and Lid. To confirm this assumption, we mixed 2 g CB with different volume of 2% Lid solution. And the obtained products were named as CB/Lid-n (n = 0, 5 and 10, indicating to the volume of Lid solution). The properties and application potential of CB/Lid-n were comprehensively investigated in vitro and in vivo.

**Fig. 2:**
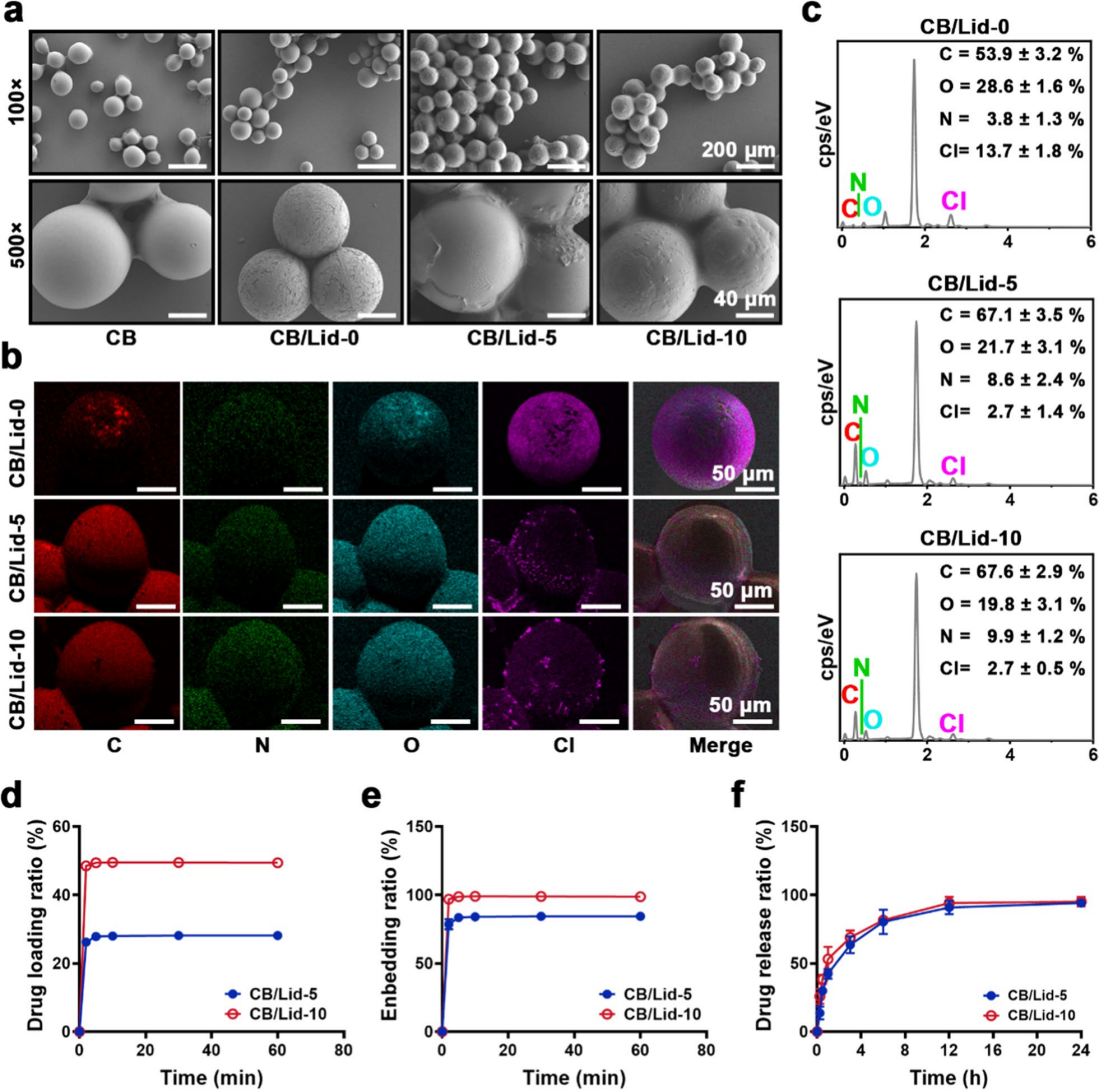
Physiochemical properties of CB/Lid-n. **a** SEM images at different magnification, scale bar: 200 or 50 µm; **b** EDS mapping of surface element distribution; **c** the content of different elements; **d** Lid-loading curves of CB/Lid-5 and CB/Lid-10; **e** Lid-enbedding curves; **f** Lid-releasing curves. Values are expressed as the mean ± SD (n = 3)

Figure 2a shows the SEM morphology of CB/Lid-n. Each group has a uniform spherical structure with a relatively smooth surface. As the n value increased from 0 to 10, the diameter of CB/Lid-n increased gradually. As shown in Additional file 1: Fig. S1, the average diameter was 96.7 ± 15.3 µm for CB group, 97.1 ± 16.9 µm for CB/Lid-0 group, 117.2 ± 16.9 µm for CB/Lid-5 group, and 124.6 ± 16.2 µm for CB/Lid-10 group. Significant difference was observed between CB/Lid-10, CB/Lid-5, and other groups (*P* < 0.05). The increased diameter was attributed to the electrostatically self-assembled Lid composites.

An EDS mapping test was also performed to visualize the Lid composites. As shown in Fig. 2b, different elements (C, N, O, and Cl) on the surface of CB/Lid-n samples were marked with different colors. CB/Lid-0 was dominated by Cl element, which mainly came from the modified components of commercial CB [38]. Lid is a clinic-used anesthetic with a chemical formula of C_14_H_22_N_2_O [39]. In CB/Lid-5 and CB/Lid-10 groups, the relative content of C and O elements obviously increased. The quantitative results of EDS mapping are shown in Fig. 2c. As the n value increased from 0 to 10, C and N elements increased, and O and Cl elements decreased. These results indicated the formation of Lid composites, which were consistent with the results of SEM.

Qualified drug carriers are required to load enough drugs and release them in a sustained manner [40]. In this study, Lid could be facilely loaded onto CB via intermolecular hydrogen bond and ionic bond. As shown in Fig. 2d, e, the equilibrium drug loading ratio was 28.16% for CB/Lid-5, and 49.36% for CB/Lid-10; the equilibrium embedding ratio was 84.48% for CB/Lid-5, and 98.73% for CB/Lid-10. The releasing dynamics of CB/Lid-n were detected in a PBS solution. As shown in Fig. 2f, CB/Lid-5 and CB/Lid-10 could release Lid for approximately 12 h, which corresponded to the most severe period of post-TAE pain. Notably, CB is reported as an ideal drug carrier of apatinib, idarubicin, arsenic trioxide, etc. [10, 41, 42]. Combined with previous literatures, we concluded that the lidocaine/CalliSpheres^®^ composites were successfully prepared, and exhibited preliminary application potential as drug carriers.

### Biocompatibility and anti-inflammatory activity of CB/Lid-n

**Fig. 3:**
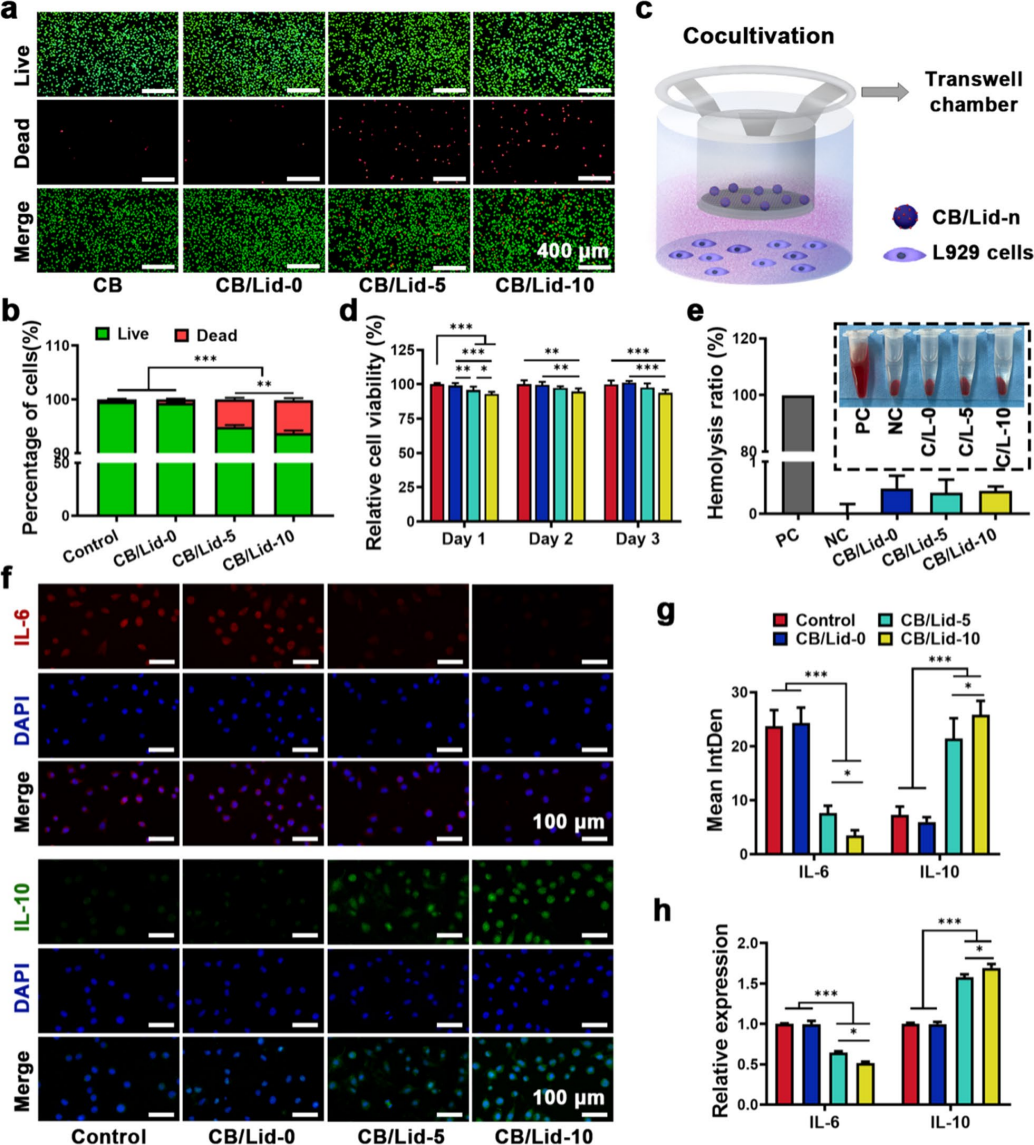
Biocompatibility and anti-inflammatory activity of CB/Lid-n. **a** Fluorescent images of live/dead staining assay, scale bar: 400 µm; **b** percentage of live cells and dead cells in each group; **c** a device diagram of modified CCK-8 assay; **d** relative cell viability of L929 cells co-cultivated with CB/Lid-n; **e** hemolysis ratio of red blood cells co-incubated with CB/Lid-n; **f** immunofluorescence images of IL-6 and IL-10, scale bar: 100 µm; **g** quantiative results of immunofluorescence images; **h** relative mRNA expression of IL-6 and IL-10. Values are expressed as the mean ± SD (n = 3). *P < 0.05, **P < 0.01, ***P < 0.001

L929 cells were used as a model cell to verify the cytocompatibility of the CB/Lid-n. The results of live/dead staining assay are shown in Fig. 3a, b. Live cells were dyed green (Calcein-AM), and dead cells were dye red (pyridine iodide). After incubated with the extracts of CB/Lid-n for 72 h, the percentage of live cells was 99.70 ± 0.14% for control group, 99.40 ± 0.16% for CB/Lid-0 group, 94.90 ± 0.32% for CB/Lid-5 group, and 93.88 ± 0.42% for CB/Lid-10 group. Almost all cells were dye green, indicating good cell viability. Meanwhile, few dead cells were found in CB/Lid-5 and CB/Lid-10 groups. This phenomenon could be attributed to the side effects of Lid [43]. The results of Live/dead staining assay were not fully satisfactory. Thus, we further performed a modified CCK-8 assay as a supplement.

As shown in Fig. 3c, CCK-8 assay were performed in a transwell chamber assay. L929 cells were seeded onto the lower chamber, and CB/Lid-n samples were added onto the upper chamber. The bioactive ingredients of CB/Lid-n could immediately move to the lower chamber. The quantitative results of CCK-8 assay are shown in Fig. 3d. The relative cell viability of CB/Lid-5 and CB/Lid-10 groups was significantly lower than that of blank control group (*P* < 0.05). Fortunately, the relative cell viability of each group was obviously higher than 80%, which are required by the U.S. Food and Drug Administration (FDA). Thus, the relatively good cytocompatibility of CB/Lid-n could meet the general requirements of biomaterials.

The hemocompatibility of CB/Lid-n was evaluated using a hemolysis test. CB/Lid-n samples were co-incubated with fresh red blood cells for 60 min, and almost no cells were lysed. The quantitative results of hemolysis rate (HR) are shown in Fig. 3e. The HR of positive control group (distilled water) and negative control group (normal saline) were set to 100% and 0%, respectively. The HR was 0.5 ± 0.2% for CB/Lid-0 group, 0.4 ± 0.3% for CB/Lid-5 group, and 0.4 ± 0.1% for CB/Lid-10 group. According to the ASTM F756-2000, HR should be no more than 5% [44]. Thus, the hemocompatibility of CB/Lid-n was proved to be excellent.

An inflammatory cell model was constructed by culturing L929 cells with 2% carrageenan medium. The treated cells were then incubated with the extracts of CB/Lid-n samples for 24 h, and the expression of IL-6 and IL-10 were detected by IF staining (Fig. 3f, g) and qRT-PCR (Fig. 3h). We found that CB/Lid-5 and CB/Lid-10 could downregulate the expression of IL-6, and upregulate the expression of IL-10. IL-6 can promote the expansion and activation of T cell population and B cell differentiation, thus aggravating the inflammatory response [45]. IL-10 is a recognized inflammatory and immunosuppressive factor [46]. Our results in vitro suggested that CB/Lid-5 and CB/Lid-10 could inhibit the carrageenan-induced inflammatory response.

### CB/Lid-n relieved pain by improving inflammatory micro-environment

**Fig. 4:**
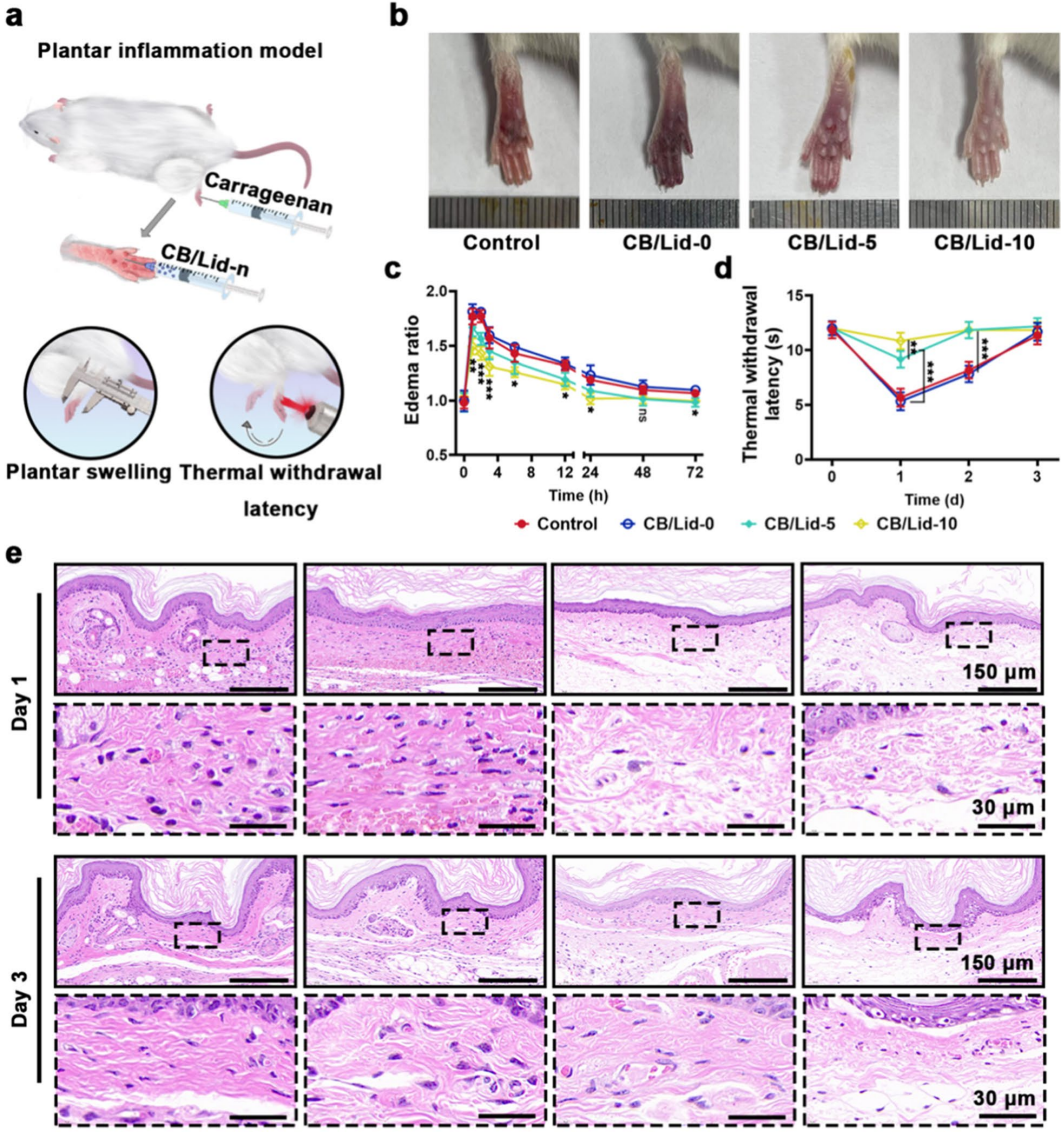
Anti-inflammatory and pain-relieving effect of CB/Lid-n in vivo. **a** Schematic illustration of plantar inflammation model; **b** optical images of injured foot; **c** dynamic changes of the plantar thickness; **d** thermal withdrawal latency; **e** HE staining images of injured foot tissue at different magnification, scale bar: 150 or 30 µm. Values are expressed as the mean ± SD (n = 3). *P < 0.05, **P < 0.01, ***P < 0.001

As shown in Fig. 4a, a mouse plantar inflammation model was constructed by injecting 100 µL carrageenan into the plantar of left hind toe. Control group was given normal saline treatment. Thus, severe inflammatory response, and even foot necrosis occurred. The other groups were given CB/Lid-n (n = 0, 5, 10) injection treatment, respectively. The optical images of treated foots are shown in Fig. 4b. Compared with control group, the foot necrosis in CB/Lid-0 group was aggravated, due to the increase of tissue tension. CB/Lid-5 and CB/Lid-10 groups showed less foot necrosis. This phenomenon might be attributed to anti-inflammatory effect of Lid. As shown in Fig. 4c, the plantar thickness (edema ratio, ER) increased in the first 1 h, and then decreased in a period of 72 h. At each time point, the growth ratio of CB/Lid-10 group was less than that of other others, which was consistent with the general observation.

Foot necrosis can be characterized by the activation of exogenous pain and temperature sensation [47]. Herein, a laser-assisted temperature sensing test was carried out according to previous report [48]. An 808 nm laser stroke the treated foots until they withdrew. As shown in Fig. 4d, the normal thermal withdrawal latency was 11.95 ± 0.62 s. At day 1, the thermal withdrawal latency was 5.67 ± 0.82 s for control group, 5.33 ± 0.82 s for CB/Lid-0 group, 9.17 ± 0.75 s for CB/Lid-5 group, and 10.83 ± 0.75 s for CB/Lid-10 group. Significant difference was observed between CB/Lid-10 group and other groups (*P* < 0.05). However, at day 3, there was no difference between CB/Lid-10 group and CB/Lid-5 group (*P* > 0.05), but significant difference was observed between those two groups and the other groups (Control group and CB/Lid-0 group, *P* < 0.01). Notably, Lid was released within the first 12 h, and it did not directly influence the temperature sensation after 24 h. In this study, temperature sensation was highly correlated with the intensity of inflammatory response.

The injured foot tissues were resected for histopathological analysis. Each group presented complete dermis and keratinized epithelium (Fig. 4e). The thickness of derma (TD) of blank control and CB/Lid-0 groups was significantly more than that of CB/Lid-5 and CB/Lid-10 groups (Additional file 1: Fig. S2a). In control group and CB/Lid-0 group, the extracellular matrix (ECM) was stained dark red with eosin, and massive inflammatory cells infiltrated. This phenomenon could be attributed to the inflammatory edema induced by carrageenan. At day 1, free red blood cells were found in the ECM of CB/Lid-0 group, suggesting that tissue necrosis occurred. In CB/Lid-5 group and CB/Lid-10 group, all the pathological changes were obviously alleviated. The mast cells were stained by toluidine blue staining assay. As shown in Additional file 1: Fig. S3, the CB/Lid-5 and CB/Lid-10 exhibited less positively stained cells, suggesting that they could inhibit the degranulation of mast cell to reduce inflammation.

**Fig. 5:**
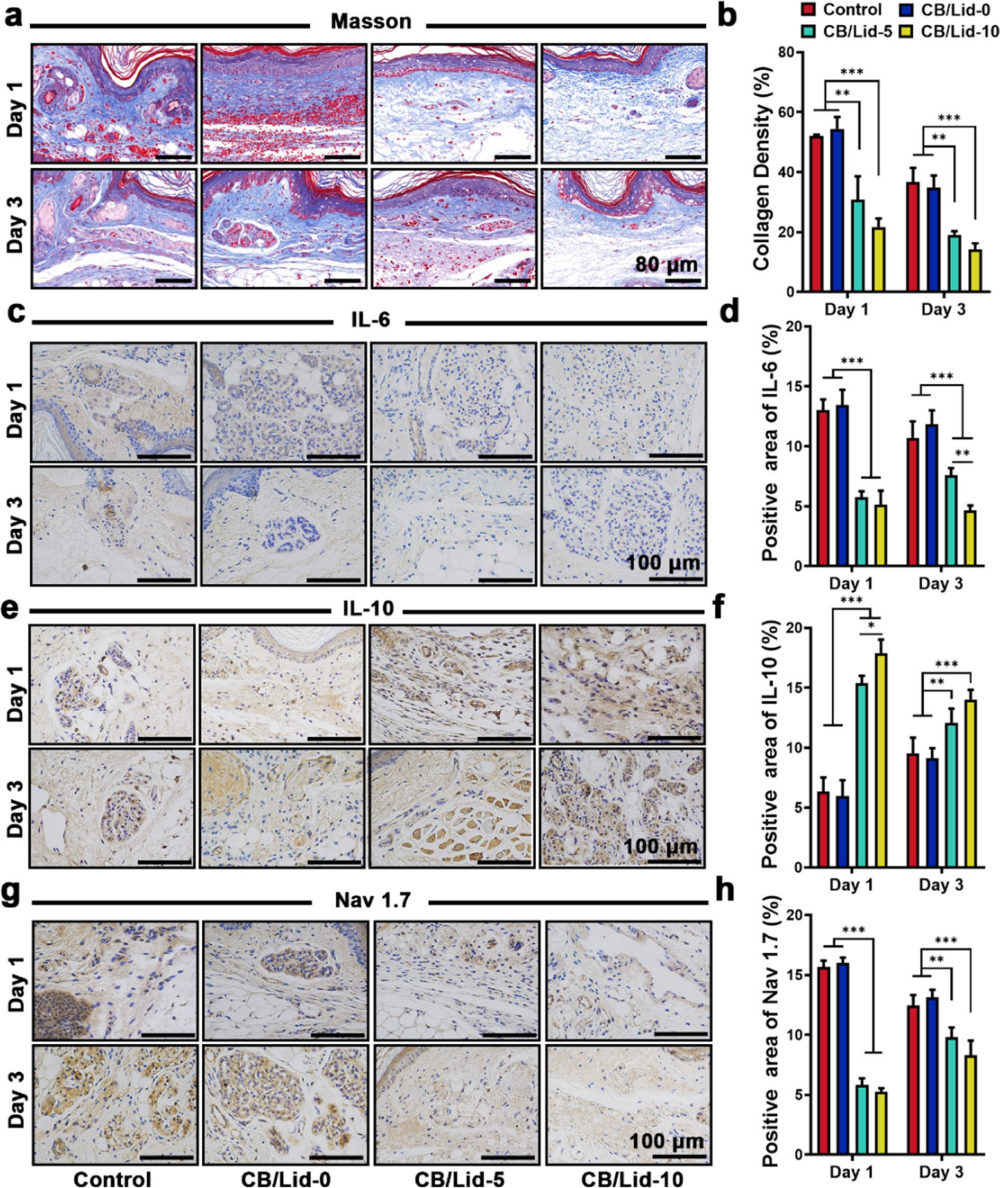
Uncovering the mechanism of CB/Lid-n via histological markers. **a**, **b** Optical images of Masson staining and the quantitative analysis, scale bar: 80 µm; **c**, **d** results of IL-6 immunohistochemical (IHC) staining, Scale bar: 100 µm; **e**, **f** results of IL-10 IHC staining, Scale bar: 100 µm; **g**, **h** results of Nav 1.7 IHC staining, Scale bar: 100 µm. Values are expressed as the mean ± SD (n = 3). **P < 0.01, ***P < 0.001

The results of Masson staining assay are shown in Fig. 5a. The red blood cells were stained red, and collagen was stained blue. At day 1, massive red blood cells were found in control group and CB/Lid-0 group, but not in CB/Lid-5 group and CB/Lid-10 group. Meanwhile, the relative content of collagen significantly decreased in CB/Lid-5 and CB/Lid-10 groups (Fig. 5b). Collagen, including collagen I and collagen III, is the main component of ECM. Under pathological conditions, host cells can release a series of cytokines (TGF-β, IL-1, IL-6, IL-10, IL-17A, TNF) to promote collagen synthesis and remodeling, and eventually induce fibrosis [49]. The harsh fibrosis process was effectively blocked by CB/Lid-5 and CB/Lid-10.

To explore the mechanism of CB/Lid-n, a series of inflammatory-related cytokines were detected by immunohistochemical (IHC) staining assay. Representative images and the quantitative results are shown in Fig. 5c–f. Compared to control group, the expression of pro-inflammatory cytokines (IL-6) was significantly down-regulated, and the expression of anti-inflammation cytokine (IL-10) were significantly up-regulated in CB/Lid-10 group (*P* < 0.001). The anti-inflammation activity of CB/Lid-10 might be directly related to IL-6 and IL-10 mediated signal pathways. To confirmed this assumption, the inflamed animals were additionally treated with recombinant IL-6 protein and anti IL-10 receptor-blocking monoclonal antibody. The results are shown in Additional file 1: Fig. S4a, b. Compared to CB/Lid-10 group, the ER of CB/Lid-10 + IL-6 group and CB/Lid-10 + anti IL-10 group increased significantly at 12 h and 24 h (*P* < 0.05), and the TWL of CB/Lid-10 + IL-6 group and CB/Lid-10 + anti IL-10 group decreased significantly at 24 h (*P* < 0.05). These results were in consistent with the assumption.

Inflammatory response is highly interacted with the generation, transduction, and processing of pain [50–53]. Generally, the releasing of cell debris and pro-inflammatory factors will inevitably generate a large amount of endogenous pain signals. Sodium channel Nav 1.7 belongs to voltage gated sodium channel (VGSC) family [54]. It is highly expressed in nociceptive neurons, and functions as sensing micro-stimulation pain signals. As shown in Fig. 5g, h, the expression of Nav 1.7 in the injured foot tissues were detected by IHC staining assay. At day 1, the positive area of Nav1.7 was 15.64 ± 0.55% for control group, 15.98 ± 0.45% for CB/Lid-0 group, 5.81 ± 0.56% for CB/Lid-5 group, and 5.23 ± 0.31% for CB/Lid-10 group; at day 3, that was 12.46 ± 0.87% for control group, 13.15 ± 0.61% for CB/Lid-0 group, 9.82 ± 0.80% for CB/Lid-5 group, and 8.30 ± 1.22% for CB/Lid-10 group. The deficiency of Nav 1.7 in CB/Lid-5 group and CB/Lid-10 group will reduce the body’s ability to perceive pain signals.

In this part, CB/Lid-5 and CB/Lid-10 could effectively improve the inflammatory micro-environment (IME), and relieve endogenous pain by inhibiting the generation and perception of pain signals. CB/Lid-5 and CB/Lid-10 could release Lid to anesthetize within the first 12 h, but relieve pain via IME-mediated pathway for a longer time. Our results suggested that the performances of CB/Lid-10 were slightly better than those of CB/Lid-5, owning to the dose-dependent effect. Thus, CB/Lid-10 was preferred for subsequent TAE therapy.

### Anti-tumor effect of CB/Lid-10

**Fig. 6:**
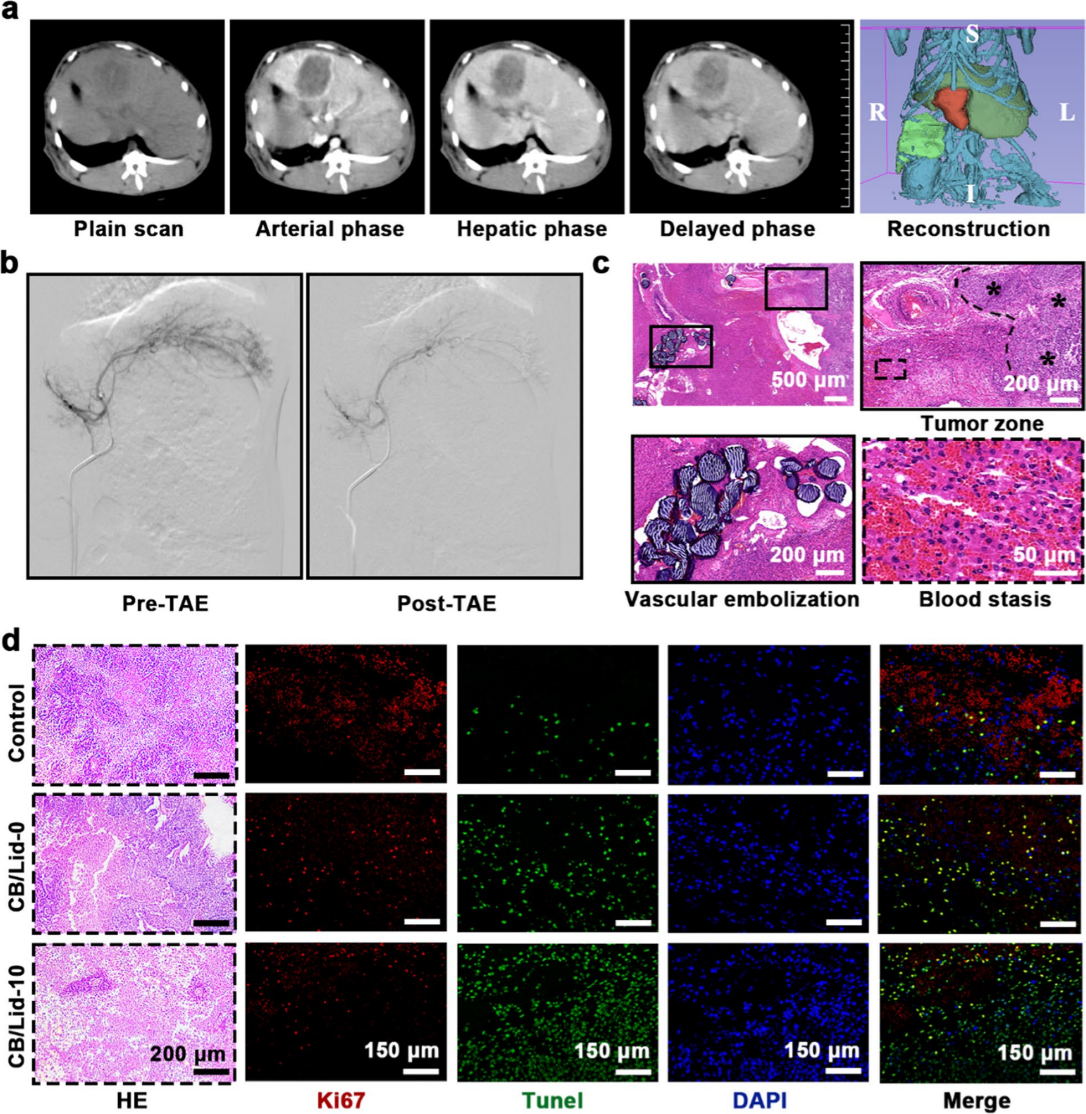
CB/Lid-10 inhibited the growth of hepatocellular carcinoma in vivo. **a** VX2-tumor bearing model were verified by CT scanning; **b** DSA images before and after TAE treatment; **c** HE staining images of embolic tissues; **d** Histological analysis of tumor tissue, including HE, Ki67 and TUNEL staining, scale bar: 200 or 150 µm. Values are expressed as the mean ± SD (n = 3). *P < 0.05

A schematic illustration of the construction process of VX2-tumor bearing model is shown in Additional file 1: Fig. S5. VX2 tissues were activated, and then transplanted into rabbit liver. The CT scanning images are shown in Fig. 6a, confirming that the animal model was successfully constructed. After that, TAE was performed to inhibit the growth of VX2 tumor. 24 h after TAE treatment, blood supply was effectively blocked by CB/Lid-10 (Fig. 6b), and the embolic tissue showed obvious ischemic necrosis (Additional file 1: Fig. S6a). The fresh liver tissue containing VX2 tumor was resected for HE analysis. As shown in Fig. 6c, CB/Lid-10 samples accumulated in the intrahepatic artery connected to tumor nidus. A large amount of red blood cells was extravasated around the tumor, cutting off the oxygen and nutrition supply. In this study, analgesic Lid but no other chemotherapeutic drug was loaded onto CB/Lid-10.

A series of histopathological analysis was performed to further verify the anti-tumor activity of CB/Lid-10. As shown in Fig. 6d, control group showed aggregated tumor cells with typical nuclear atypia. The number of tumor cells was significantly decreased in CB/Lid-0 and CB/Lid-10 groups, owning to the effect of embolization. Ki67 is one of the protein markers of cell proliferation, and TUNEL is an effective method to label apoptotic cells [32]. As shown in Additional file 1: Fig S6b, c, the Ki67 positively stained area was 15.62 ± 1.03% for control group, 8.25 ± 0.43% for CB/Lid-0 group, and 4.01 ± 0.55% for CB/Lid-10 group; apoptotic index was 17.65 ± 1.28% for control group, 61.92 ± 9.07% for CB/Lid-0 group, and 93.96 ± 2.55% for CB/Lid-10 group. It was concluded that CB/Lid-0 and CB/Lid-10 could inhibit cell proliferation and promote cell apoptosis. Meanwhile, CB/Lid-0 and CB/Lid-10 could also inhibit lung metastasis of VX2 tumor (Additional file 1: Fig. S6d). Notably, the anti-tumor activity of CB/Lid-10 was significantly better than that of CB/Lid-0, suggesting that Lid-mediated inflammation modulation might play an auxiliary role in TAE treatment.

As shown in Additional file 1: Fig. S7a, CB/Lid-10 samples were intra-arterially injected into liver tissue, and then released Lid into blood in a sustained manner. Lid is slowly excreted by the kidney, and the excess Lid in the blood may lead to central nervous system damage [55, 56]. As shown in Additional file 1: Fig. S7b, the plasma concentration of Lid was monitored. It was 1.51 ± 1.63 µg/mL immediately after TAE treatment, owning to the burst-releasing of Lid. The plasma concentration of Lid was quickly diluted by blood flow within 30 min, and reached to a relatively safe level. Thus, the TAE treatment of CB/Lid-10 will not lead to systemic side effects.

**Fig. 7:**
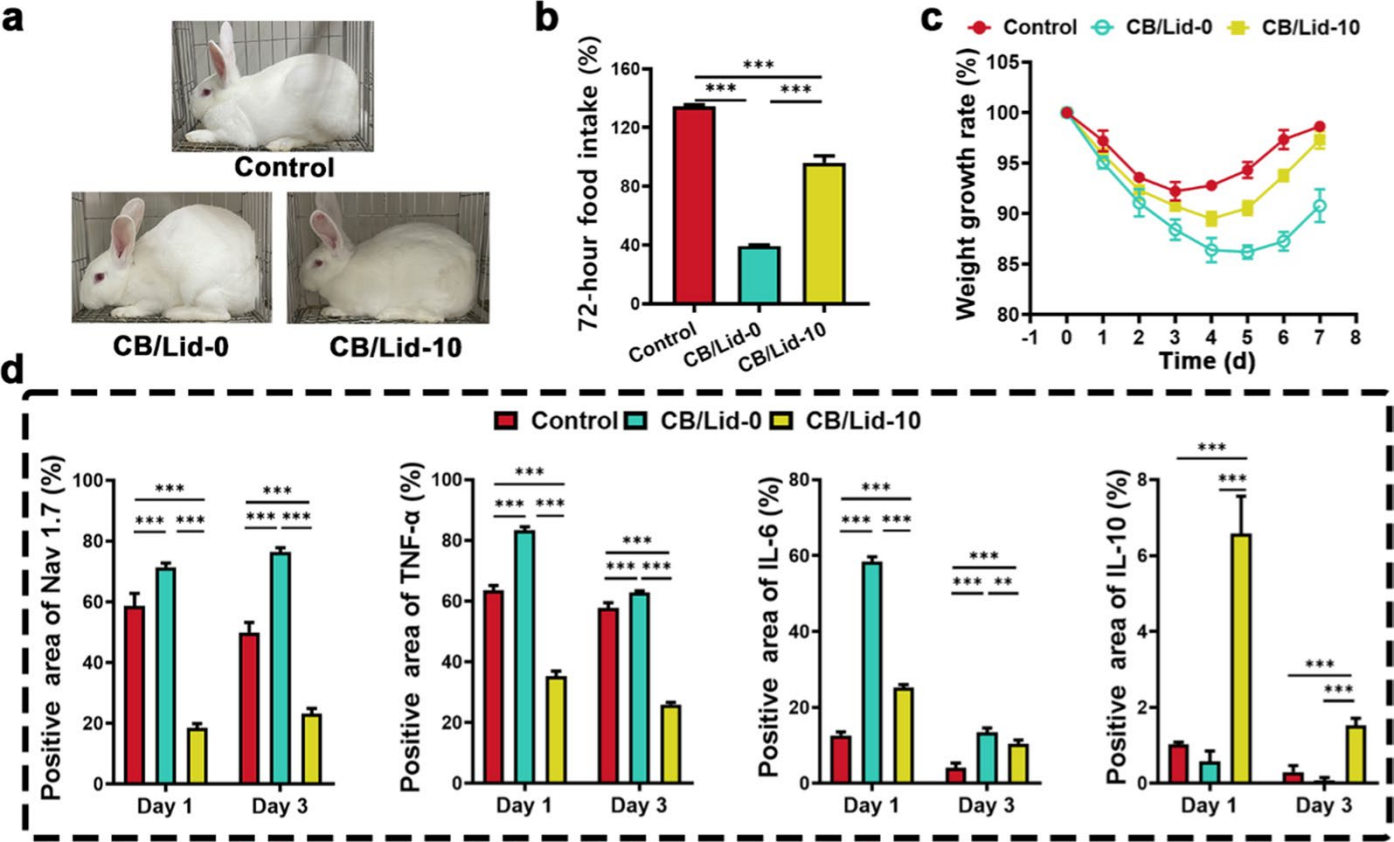
CB/lid-10 inhibited TAE-induced pain via inflammation-mediated pathway. **a** Optical images of postoperative animals; **b** 72 h food intake; **c** growth curves of body weight; **d** the protein expression of Nav 1.7, TNF-α, IL-6 and IL-10, which were quantitatively analyzed from the IHC staining images. Values are expressed as the mean ± SD (n = 3). ***P < 0.001

The pain-relieving effect of CB/Lid-10 was observed by a series of behavioral indicators. As shown in Fig. 7a, the control group was injected with normal saline, and no obvious arched back occurred. The degree of arched back of CB/Lid-0 was obviously higher than that of control group, owning to TAE-induced pain. Meanwhile, The CB/Lid-10 group showed a more comfortable posture than CB/Lid-0 group, indicating that the Lid released by CB/Lid-10 could effectively alleviate the pain. At day 3, the food intake ratio of control group increased to 134.35 ± 1.14%, and that was 39.34 ± 0.70% for CB/Lid-0 group, and 96.15 ± 4.61% for CB/Lid-10 group (Fig. 7b). Significant difference was observed between CB/Lid-0 and CB/Lid-10 groups (*P* < 0.001). Body weight of treated animals decreased gradually within post-operative 4 days, and then increased slowly (Fig. 7c). Compared to CB/Lid-0 group, the loss of body weight of CB/Lid-10 group was obviously improved. An evaluation-criteria of pain in experimental rabbit are shown in Additional file 1: Table S3. Based on these qualitative and quantitative results, we safely concluded that CB/Lid-10 could effectively alleviate the TAE-induced pain by the release of Lid.

The pain-relieving effect of CB/Lid-10 was attributed to the inflammation-mediated pathway. As shown in Additional file 1: Fig. S8–11, liver tissue adjacent to tumor was resected for immunohistochemical staining assay. A series of protein markers, such as Nav 1.7, TNF-α, IL-6, and IL-10 were detected. The quantitaitive results are shown in Fig. 7d. Compared to CB/Lid-0 group, the CB/Lid-10 group downregulated the expression of pain generator (Nav 1.7) and pro-inflammation cytokines (TNF-α and IL-6), and up-regulated thr expression of anti-inflammation cytokine (IL-10). Significant difference was observed between CB/Lid-0 and CB/Lid-10 groups (*P* < 0.001). These results were in consistent with those of plantar inflammation model.

In this study, CB/Lid-10 was successfully applied into painless TAE treatment of VX2 tumor. CB/Lid-10 could inhibit tumor cell proliferation, and induce tumor cell apoptosis via blocking the local blood supply. Furthermore, CB/Lid-10 could also alleviate the TAE-induced pain by releasing Lid. The remodeling effect of Lid on the pain generator and harsh inflammation micro-environment was confirmed. CB/Lid-10 was biocompatible, and showed great potential for clinical transformation.

## Conclusions

Herein, a series of Lidocaine-loaded CalliSpheres^®^ composites were fabricated using an electrostatic self-assembly technique. The products (CB/Lid-n) exhibited good drug-loading ratio (28.16–49.36%), drug-embedding ratio (84.48–98.73%), and sustained-release ability for almost 12 h. CB/Lid-n had relatively good cytocompatibility and hemocompatibility, which meets the general requirements of biomedical devices. CB/Lid-n could also improve carrageenan-induced inflammation micro-environment in vivo and in vitro, and then relieved endogenous pain by blocking the generation and perception of pain signals. CB/Lid-10 was firstly applied into painless TAE treatment in a VX2-tumor bearing model, and received great success. Compared to systemic administration, CB/Lid-10 exhibited great advantages in prolonging the onset time and reducing side effects. This study is of great significance for the functional modification of commercial medical devices.

## Supplementary Information

**Additional file 1: Table S1.** List of antibodies used in this work. **Table S2.** List of primers used for qRT-PCR analysis.** Table S3. **Assessment of pain in rabbit model.** Fig. S1.** Diameter distribution of CB and CB/Lid-n (n = 0, 5, 10). **Fig. S2. **(a) The thickness of derma and (b) collagen density in plantar inflammation model. Values are expressed as the mean ± SD (n = 3). Compared to control group, *P < 0.05, **P < 0.01, ***P < 0.001.** Fig. S3**. Results of toluidine blue staining assay, Scale bar: 200 µm.** Fig. S4.** (a) Dynamic changes of the edema ratio; (d) Dynamic changes of thermal withdrawal latency. Values are expressed as the mean ± SD (n = 3). *P< 0.05, **P< 0.01, ***P< 0.001. **Fig. S5.** (a) The construction process of VX2-tumor bearing model; (b) VX2-tumor tissues were successfully transplanted into the liver of rabbits.** Fig. S6.** (a) Optical images of liver tissues immediately after TAE treatment; (b) Quantitative results of Ki67 immunofluorescence staining assay, and the percentage of area positive for fluorescence was manually counted; (c) Quantitative results of TUNEL assay; (d) HE staining images of lung tissue with metastatic tumor lesions. Scale bar: 500 µm. Values are expressed as the mean ± SD (n = 3). **P< 0.01, ***P< 0.001.** Fig. S7. **(a) Schematic illustration of TAE trestment; (b) Plasma concentration of Lid.** Fig. S8. **Immunohistochemical images of Nav 1.7 at different magnification. Scale bar: 300 or 60 µm.** Fig. S9. **Immunohistochemical images of TNF-α at different magnification. Scale bar: 300 or 60 µm.** Fig. S10. **Immunohistochemical images of IL-6 at different magnification. Scale bar: 300 or 60 µm.** Fig. S11. **Immunohistochemical images of IL-10 at different magnification. Scale bar: 300 or 60 µm.

## Data Availability

Datasets analyzed and/or generated during the current study are not publicly available due to confidentiality agreements but are available from the corresponding author upon reasonable request.

## Abbreviations

TAE: Transcatheter arterial embolization
CB: CalliSpheres^®^ bead
qRT-PCR: Quantitative real-time PCR
IHC: Immunohistochemical
TWL: Thermal withdrawal latency
ER: Edema ratio
IME: Inflammation micro-environment
HCC: Hepatocellular carcinoma
MIIT: Minimally invasive interventional therapy
JSH: Japan Society of Hepatology
PVA: Polyvinyl alcohol
DRG: Diagnosis related groups
FDA: Food and drug administration
NMPA: National medical products administration
NSAID: Nonsteroidal anti-inflammatory drugs
SEM: Scanning electron microscope
DMSO: Dimethyl sulfoxide
PFA: Paraformaldehyde
HR: Hemolysis rate
SPF: Specific pathogen free
TD: Thickness of derma
ECM: Extracellular matrix
VGSC: Voltage gated sodium channel
