# Supplementary material for: One-step fabrication of lidocaine/CalliSpheres® composites for painless transcatheter arterial embolization
Source: J Transl Med. 2022 Oct 11;20:463. doi: 10.1186/s12967-022-03653-8 (PMC9552470; doi:10.1186/s12967-022-03653-8)
Supplement: Supplementary file 1 — Additional file 1: Table S1. List of antibodies used in this work. Table S2. List of primers used for qRT-PCR analysis. Table S3. Assessment of pain in rabbit model. Fig. S1. Diameter distribution of CB and CB/Lid-n (n = 0, 5, 10). Fig. S2. (a) The thickness of derma and (b) collagen density in plantar inflammation model. Values are expressed as the mean ± SD (n = 3). Compared to control group, *P < 0.05, **P < 0.01, ***P < 0.001. Fig. S3. Results of toluidine blue staining assay, Scale bar: 200 µm. Fig. S4. (a) Dynamic changes of the edema ratio; (d) Dynamic changes of thermal withdrawal latency. Values are expressed as the mean ± SD (n = 3). *P< 0.05, **P< 0.01, ***P< 0.001. Fig. S5. (a) The construction process of VX2-tumor bearing model; (b) VX2-tumor tissues were successfully transplanted into the liver of rabbits. Fig. S6. (a) Optical images of liver tissues immediately after TAE treatment; (b) Quantitative results of Ki67 immunofluorescence staining assay, and the percentage of area positive for fluorescence was manually counted; (c) Quantitative results of TUNEL assay; (d) HE staining images of lung tissue with metastatic tumor lesions. Scale bar: 500 µm. Values are expressed as the mean ± SD (n = 3). **P< 0.01, ***P< 0.001. Fig. S7. (a) Schematic illustration of TAE trestment; (b) Plasma concentration of Lid. Fig. S8. Immunohistochemical images of Nav 1.7 at different magnification. Scale bar: 300 or 60 µm. Fig. S9. Immunohistochemical images of TNF-α at different magnification. Scale bar: 300 or 60 µm. Fig. S10. Immunohistochemical images of IL-6 at different magnification. Scale bar: 300 or 60 µm. Fig. S11. Immunohistochemical images of IL-10 at different magnification. Scale bar: 300 or 60 µm. [file 12967_2022_3653_MOESM1_ESM.docx]

**One-step fabrication of** **lidocaine/****CalliSpheres® composites for painless** **transcatheter arterial embolization**

Chuan Tian^a,1^, Zijian Wang^b,1^, Lei Huang^c^, Yimin Liu^a^, Kunpeng Wu^a^, Zhaonan Li^a^, Bin Han^b^, Dechao Jiao^a^, Xinwei Han^a,*^ and Yanan Zhao^a,*^

^a^ Department of Interventional Radiology, The First Affiliated Hospital of Zhengzhou University, Zhengzhou 450052, China

^b^ Department of Urology, Zhongnan Hospital of Wuhan University, Wuhan 430071, China

^c^ Department of Plastic Surgery, Zhongnan Hospital of Wuhan University, Wuhan 430071, China

^d^ Department of Radiotherapy, The First Affiliated Hospital of Zhengzhou University, Zhengzhou 450052, China

*Corresponding authors at: Department of Interventional Radiology, The First Affiliated Hospital of Zhengzhou University, Zhengzhou 450052, China; E-mail address: [fcchanxw@zzu.edu.cn](mailto:fcchanxw@zzu.edu.cn) (X. Han); [yananzhao996@163.com](mailto:yananzhao996@163.com) (Y. Zhao).

^1^These authors contributed equally to this work.

**Table S1**. List of antibodies used in this work

| **Vendor** | **Antibody** | **Catalog no.** | **Working dilution** |
| --- | --- | --- | --- |
| Bioss | Rabbit anti-Nav 1.7 | Bs-21384R | IHC, 1:200 |
| Servicebio | Rabbit anti-IL-6 | GB11117 | IHC, 1:500 |
| Servicebio | Rabbit anti-IL-10 | GB11108 | IHC, 1:500 |
| Servicebio | Rabbit anti-TNF-α | GB11188 | IHC, 1:500 |
| Servicebio | Rabbit anti-Ki67 | GB121141 | IF, 1:200 |
| Servicebio | Goat anti-rabbit secondary antibody | G1213 | IHC, 1:200 |
| Servicebio | Cy3 conjugated Goat Anti-mouse IgG (H+L) | GB21301 | IF, 1:300 |

**Table S2.** List of primers used for qRT-PCR analysis

| **Accession Number** | **Target gene** | **Forward**  **(5’-3’)** | **Reverse**  **(5’-3’)** | **Size (bp)** | **Tm (℃)** |
| --- | --- | --- | --- | --- | --- |
| NM_031168.2 | IL-6 | CTGGGGATGTCTGTAGCTCA | CTGTGAAGTCTCCTCTCCGG | 176 | 58.5 |
| NM_010548.2 | IL-10 | GGTGAGAAGCTGAAGACCCT | TGTCTAGGTCCTGGAGTCCA | 247 | 59.0 |
| NM_133289.2 | Nav 1.7 | AGTGGTTATGGCAGCAGTCT | GAGGGTTGTCAACAGTGCTG | 222 | 59.0 |
| NM_008084.3 | GAPDH | CAACTCCCACTCTTCCACCT | GAGTTGGGATAGGGCCTCTC | 204 | 58.9 |

**Table S3.** Assessment of pain in rabbit model

| **Behavior** | **Mild pain** | **Moderate pain** | **Severe pain** |
| --- | --- | --- | --- |
| Arching | Short | Intermittent | Continuous |
| Food intake | Only 40-75% of normal intake within 72 hours | Less than 40% of normal intake within 72 hours | Less than 40% of normal intake within 7 days, or inappetence more than 72 hours |
| Bodyweight change | Weight loss less than 10% | Weight loss around 10% to 25% | Weight loss more than 75% |


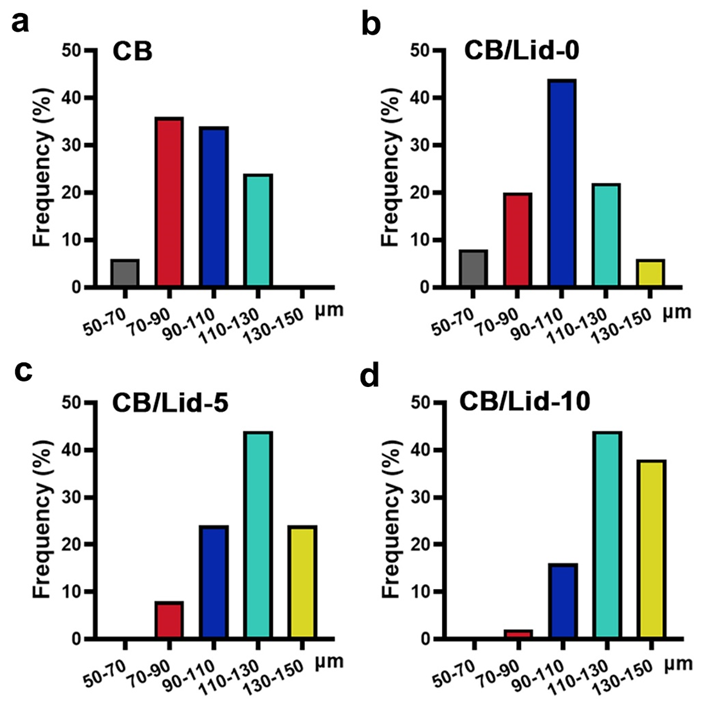


**Fig. S1.** Diameter distribution of CB and CB/Lid-n (n = 0, 5, 10).


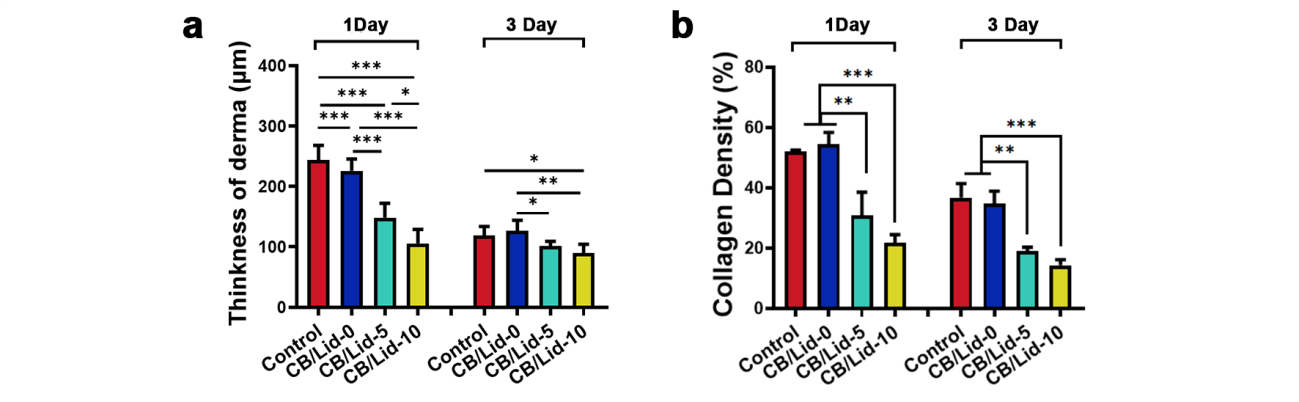


**Fig. S2.** (a) The thickness of derma and (b) collagen density in plantar inflammation model. Values are expressed as the mean ± SD (n = 3). Compared to control group, **P* < 0.05, ***P* < 0.01, ****P* < 0.001.


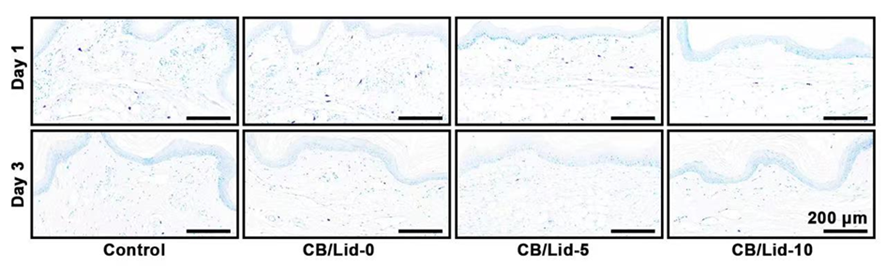


**Fig. S3**. Results of toluidine blue staining assay, Scale bar: 200 µm


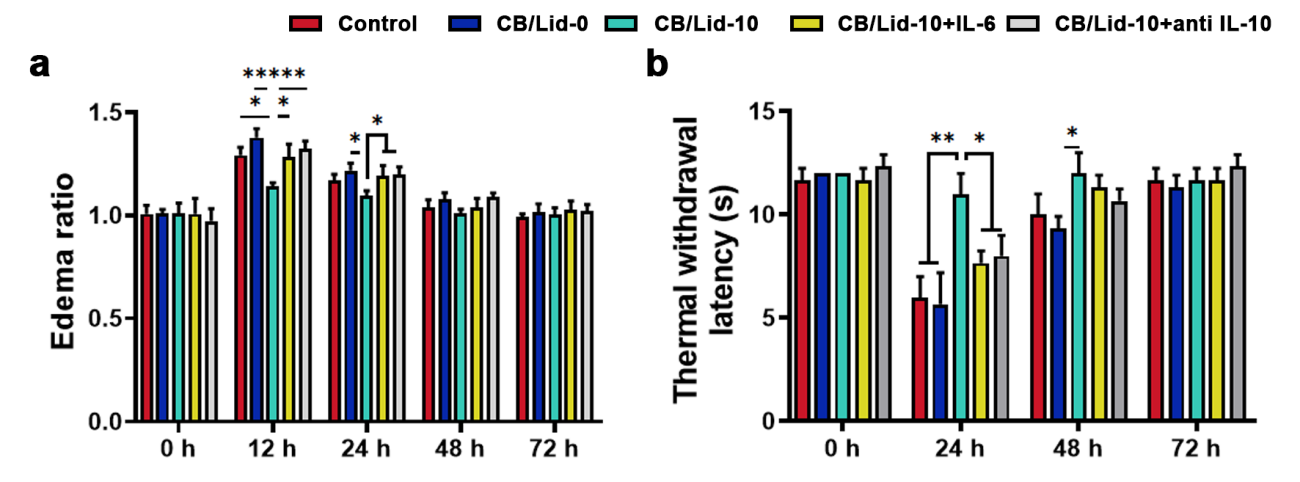


**Fig. S4.** (a) Dynamic changes of the edema ratio; (d) Dynamic changes of thermal withdrawal latency. Values are expressed as the mean ± SD (n = 3). **P*< 0.05, ***P*< 0.01, ****P*< 0.001.


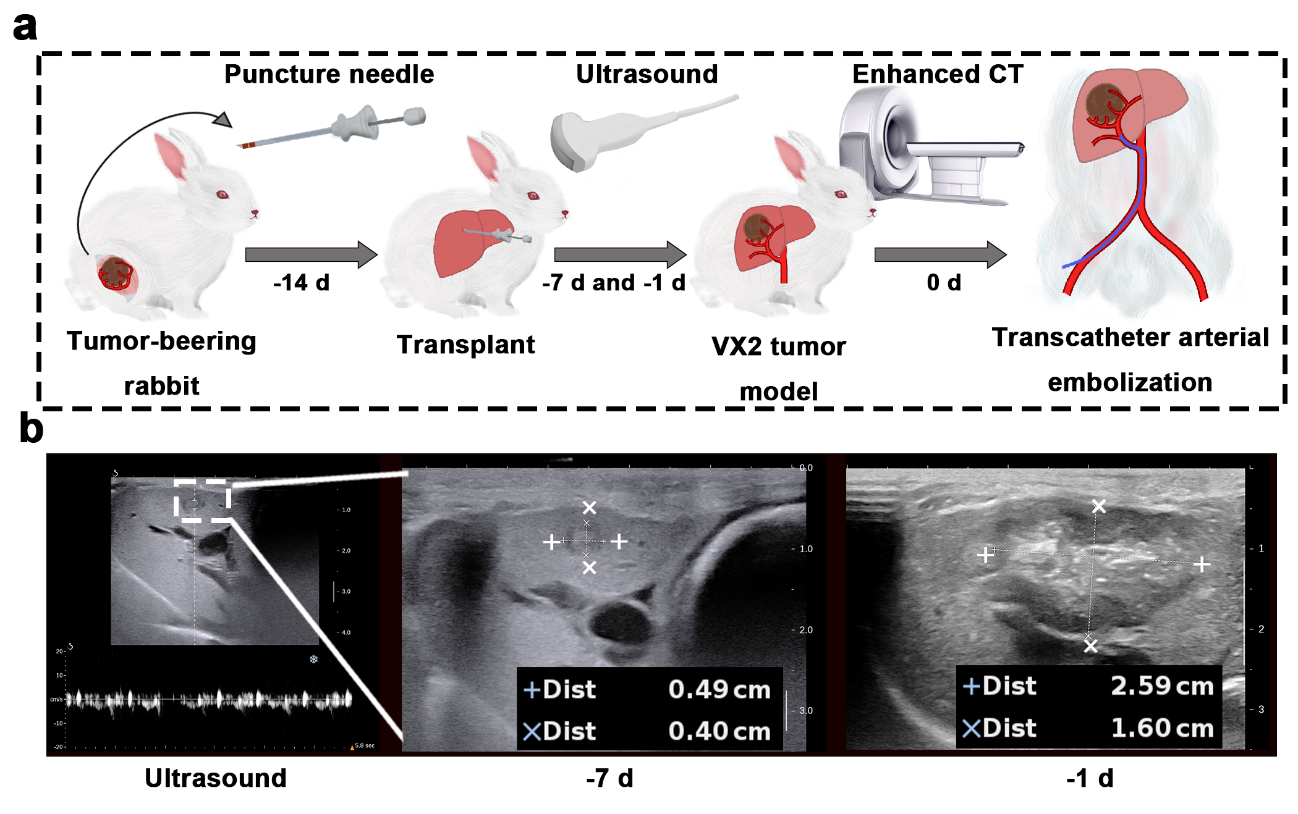


**Fig. S5.** (a) The construction process of VX2-tumor bearing model; (b) VX2-tumor tissues were successfully transplanted into the liver of rabbits.


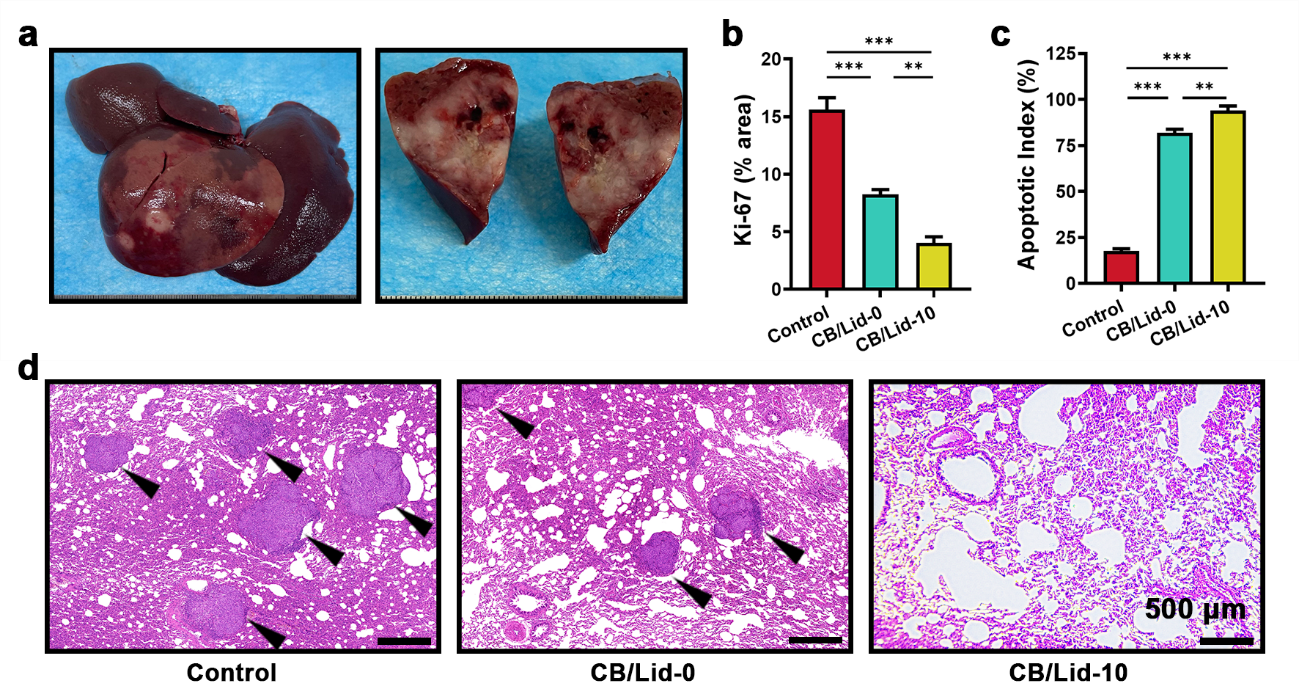


**Fig. S6.** (a) Optical images of liver tissues immediately after TAE treatment; (b) Quantitative results of Ki67 immunofluorescence staining assay, and the percentage of area positive for fluorescence was manually counted; (c) Quantitative results of TUNEL assay; (d) HE staining images of lung tissue with metastatic tumor lesions. Scale bar: 500 µm. Values are expressed as the mean ± SD (n = 3). ***P*< 0.01, ****P*< 0.001.

**
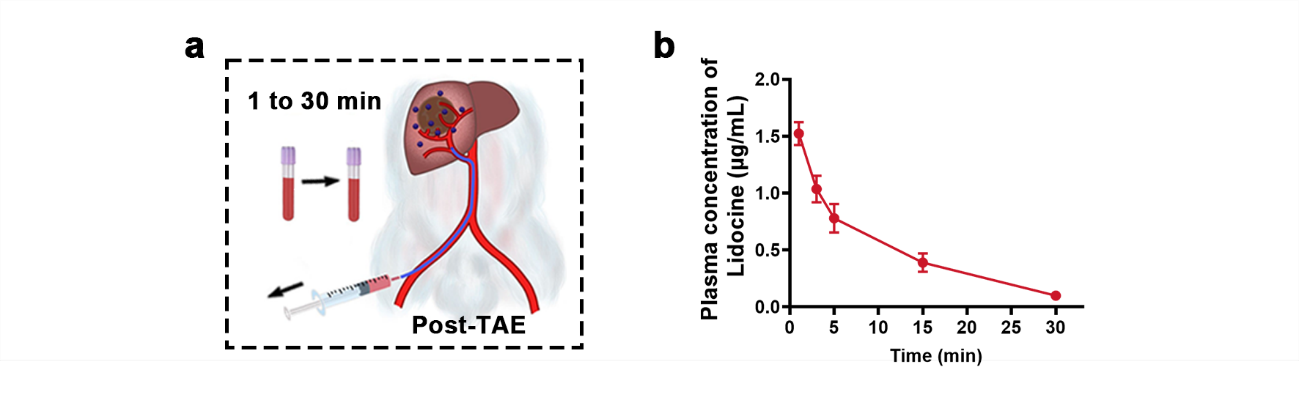
**

**Fig. S7.** (a) Schematic illustration of TAE trestment; (b) Plasma concentration of Lid.


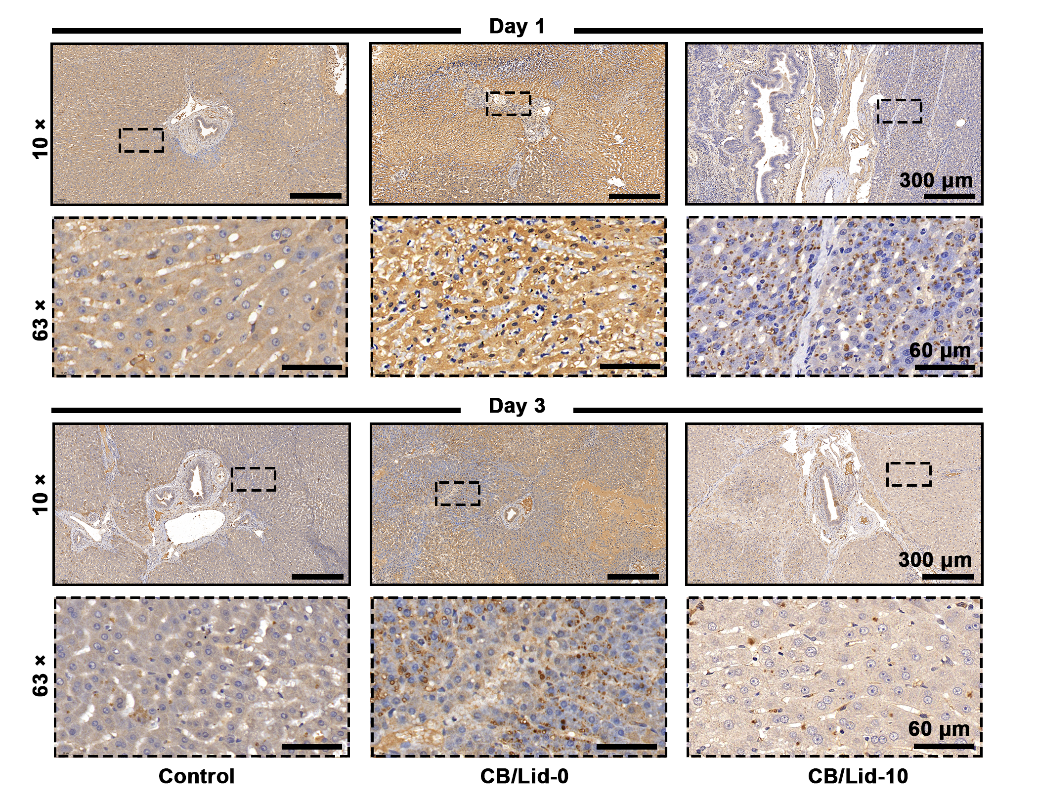


**Fig. S8.** Immunohistochemical images of Nav 1.7 at different magnification. Scale bar: 300 or 60 µm.


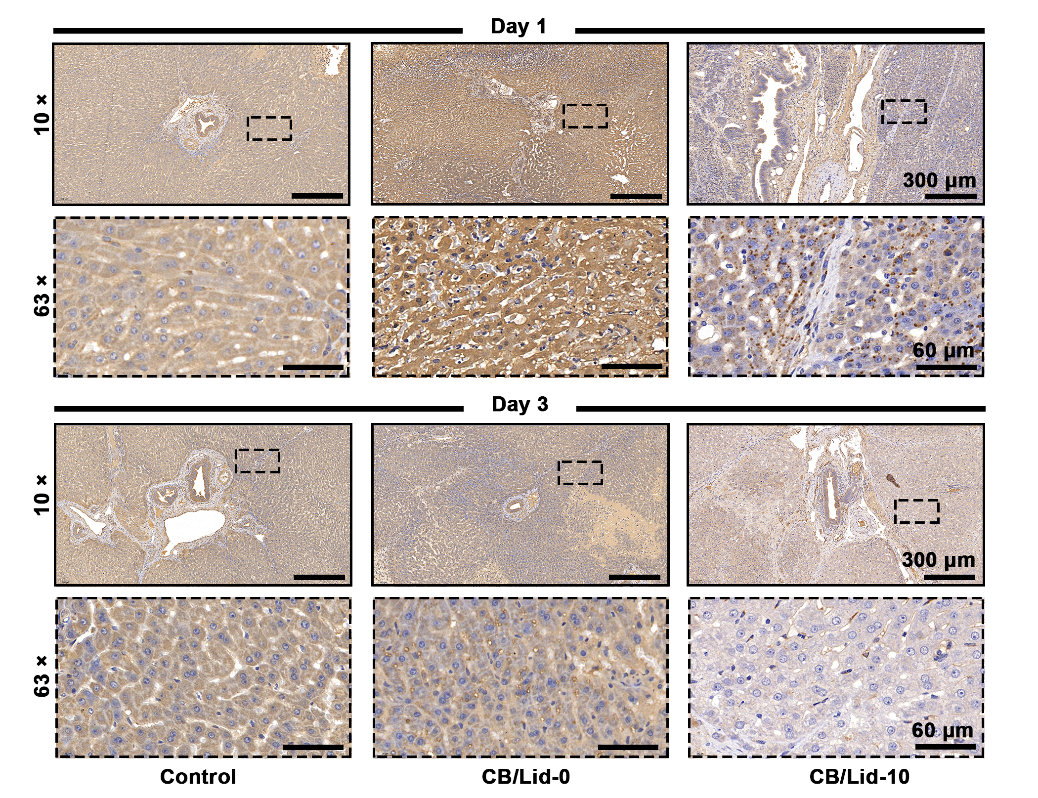


**Fig. S9.** Immunohistochemical images of TNF-α at different magnification. Scale bar: 300 or 60 µm.


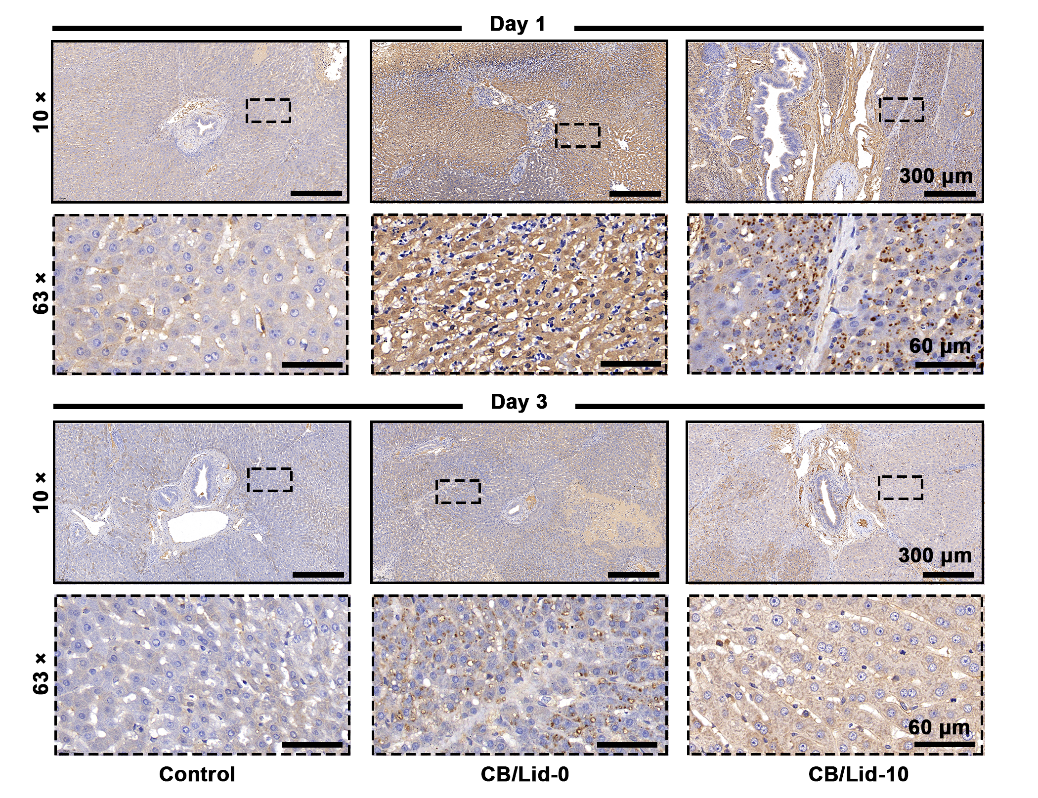


**Fig. S10.** Immunohistochemical images of IL-6 at different magnification. Scale bar: 300 or 60 µm.


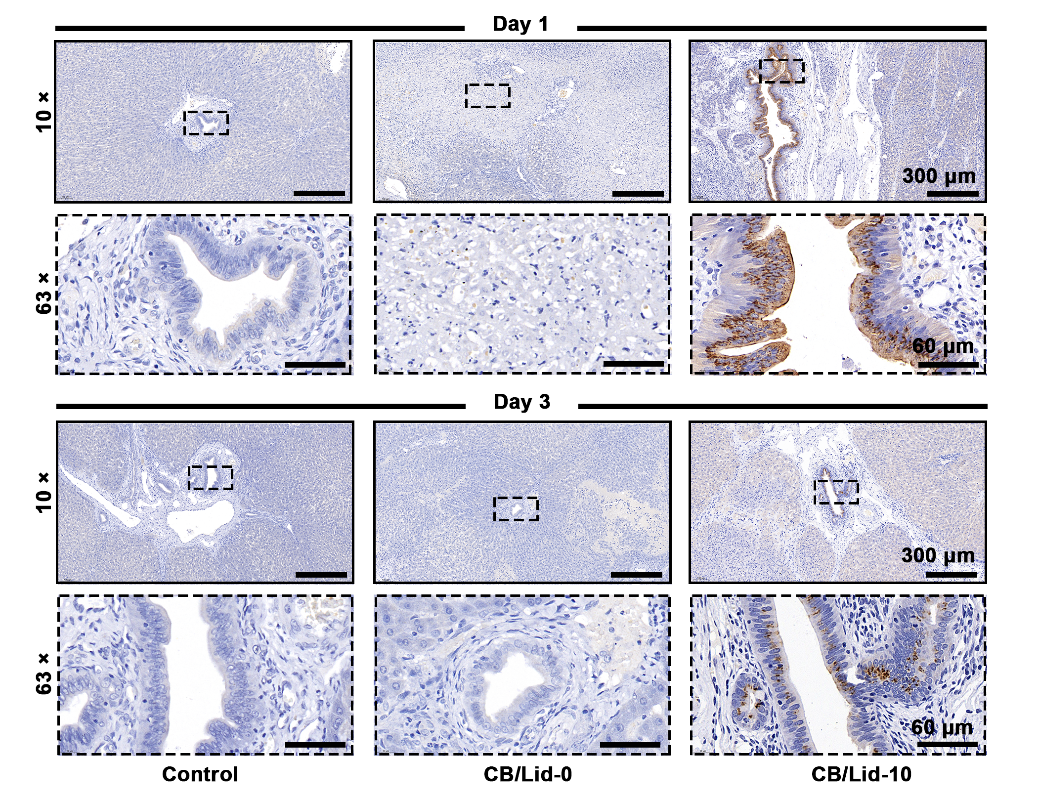


**Fig. S11.** Immunohistochemical images of IL-10 at different magnification. Scale bar: 300 or 60 µm.
